# Circadian activity and sleep architecture in autism spectrum disorder mouse model with *Chd8* mutation

**DOI:** 10.3389/frsle.2025.1614100

**Published:** 2025-08-06

**Authors:** Jiahui Yu, Norie Deki-Arima, Yuki C. Saito, Naoki Furutani, Masaaki Nishiyama, Keiichi I. Nakayama, Yasutaka Niwa, Arisa Hirano, Takeshi Sakurai

**Affiliations:** ^1^International Institute for Integrative Sleep Medicine (WPI-IIIS), Tsukuba Institute for Advanced Research (TIAR), University of Tsukuba, Tsukuba, Ibaraki, Japan; ^2^Degree Program in Comprehensive Human Sciences, Graduate School of Comprehensive Human Science, University of Tsukuba, Tsukuba, Ibaraki, Japan; ^3^Institute of Medicine, University of Tsukuba, Tsukuba, Ibaraki, Japan; ^4^Department of Psychiatry and Cognitive-Behavioral Medicine, Nagoya City University Graduate School of Medical Sciences, Nagoya, Japan; ^5^Social Brain Development Research Unit, Next Generation Medical Development Research Core, Institute for Frontier Science Initiative, Kanazawa University, Kanazawa, Ishikawa, Japan; ^6^Department of Histology and Cell Biology, Graduate School of Medical Sciences, Kanazawa University, Kanazawa, Ishikawa, Japan; ^7^Anticancer Strategies Laboratory, Advanced Research Initiative, Institute of Science Tokyo, Tokyo, Japan; ^8^Department of Pharmacology, Biomedical Research Center, Hirosaki University Graduate School of Medicine, Hirosaki, Aomori, Japan; ^9^Life Science Center for Tsukuba Advanced Research Alliance, University of Tsukuba, Tsukuba, Ibaraki, Japan

**Keywords:** autism spectrum disorder (ASD), sleep, EEG, *CHD8*, *Chd8* knockout mice

## Abstract

Individuals with autism spectrum disorder (ASD) frequently experience sleep disturbances, including difficulties in sleep initiation, reduced total sleep time, and excessive daytime sleepiness. Among them, those carrying mutations in the *CHD8*, a high-penetrance ASD risk gene, often exhibit both core ASD symptoms and pronounced sleep abnormalities. However, detailed evaluations of sleep architecture and circadian activity in this population remain limited. In this study, we characterized the daily sleep patterns of *Chd8* heterozygous knockout mice of both sexes, an established ASD model, using electroencephalography (EEG)/electromyography (EMG) recordings. *Chd8* knockout mice exhibited reduced wakefulness and increased rapid eye movement (REM) sleep duration during the dark phase, along with disruption of normal daily REM sleep fluctuations. Furthermore, analysis of REM latency distributions revealed a reduction in short-latency REM bouts (i.e., <150 seconds) during the light phase. *Chd8* knockout also showed reduced locomotor activity at night. These findings provide new insights into the sleep phenotypes associated with *CHD8*-related ASD and may help elucidate the underlying neurobiological mechanisms of sleep disturbances in this condition.

## 1 Introduction

Autism spectrum disorder (ASD) is an early-onset neurodevelopmental condition, typically beginning in childhood, with a prevalence of approximately 1% and a male-to-female ratio of 4.2 (Zeidan et al., 2022). According to the Diagnostic and Statistical Manual of Mental Disorders, 5th Edition (DSM-5), ASD is characterized by persistent deficits in social communication and interaction, as well as restricted and repetitive behaviors and interests, all of which impair daily functioning.

Sleep disturbances are a common comorbidity in ASD, affecting 40% to 80% of individuals and frequently manifesting as insomnia (Cohen et al., 2014; Mannion and Leader, 2014; Chen et al., 2021). Studies have shown that younger children (3–7 years old) with ASD often experience bedtime resistance, sleep anxiety, and frequent night awakenings (Goldman et al., 2012; Hodge et al., 2014; Galli et al., 2022), whereas older children and adolescents (≥7 years old) are more prone to delayed sleep onset, reduced sleep duration, and excessive daytime sleepiness (Goldman et al., 2012; Hodge et al., 2014; Galli et al., 2022). For example, Galli et al. reported that 37% of ASD children exhibit excessive daytime sleepiness, with 68% of them sleeping for two or more hours during the day (Galli et al., 2022). Importantly, these sleep problems often persist across the lifespan and do not resolve with age (Goldman et al., 2011, 2012; Hodge et al., 2014). There is growing evidence that sleep quality is closely linked to daytime behaviors in individuals with ASD (Goldman et al., 2011; Cohen et al., 2014; Mannion and Leader, 2014; Chen et al., 2021; Galli et al., 2022). Sleep disturbance is thought to contribute bidirectionally to ASD symptoms (Cohen et al., 2014). For instance, poor sleep has been associated with greater severity of core symptoms, such as restricted and repetitive behaviors and impairments in social communication (Goldman et al., 2011; Cohen et al., 2014). Conversely, improving sleep quality through parent education, behavioral interventions, or pharmacological treatments has been shown to alleviate ASD symptoms and improve daytime functioning (Malow et al., 2012; Cohen et al., 2014; Buckley et al., 2020; Galli et al., 2022). Therefore, understanding the nature and mechanisms of sleep problems in individuals with ASD may offer valuable insights for developing more effective interventions.

ASD is a highly heterogeneous disorder with a complex genetic architecture, involving not only common mutations but also rare mutations and de novo mutations (Varghese et al., 2017; Lord et al., 2020; Wintler et al., 2020; Jiang et al., 2022). Using ASD mouse models with gene mutations or deletions allows for a detailed exploration of sleep patterns and circadian activities. However, considerable variability in sleep phenotypes has been reported across different ASD mouse models in previous studies (Wintler et al., 2020; Maurer et al., 2023). Some models have shown increased wakefulness. For example, mice with 16p11.2 deletion or *Ctnnd2* knockout mice exhibited increased total wakefulness and decreased non-rapid eye movement sleep (NREM) and rapid eye movement sleep (REM) (Angelakos et al., 2017; Lu et al., 2018; Xu et al., 2023), while mice with a mutation in *Scn1a* gene or *Mecp2* knockout mice showed increased wakefulness only during the dark phase (Papale et al., 2013; Johnston et al., 2014). Another study observed no changes in total sleep duration but observed abnormal EEG spectral patterns during both NREM sleep and REM sleep, as well as reduced spindles in *Scn1a* mutant mice compared to controls (Kalume et al., 2015). Additionally, mice with *Cacna1h* mutations did not show altered wakefulness, but exhibited a decrease in total sleep time (Tatsuki et al., 2016). In contrast, studies involving mice with mutations in *Shank3* or *Csnk1e* have reported increased REM sleep during the dark phase despite increased wakefulness (Zhou et al., 2014; Medina et al., 2022). These phenotypic differences are likely attributable to the functional heterogeneity of ASD-related genes, which are involved in diverse biological processes including synaptic scaffolding (*Shank3*), transcriptional regulation (*Mecp2*), cell adhesion (*Ctnnd2*), signal transduction (*Csnk1e*), and ion channel activity (*Scn1a* and *Cacna1h*). These mutations are thought to disrupt synaptic plasticity, neuronal morphology, and the excitatory-inhibitory balance within cortical circuits (Ingiosi et al., 2019; Takumi et al., 2020). Despite growing knowledge, the precise molecular mechanisms and neural circuits underlying sleep phenotypes remain largely elusive. These mutations may also affect broader biological processes, including neurodevelopment and circadian regulation, thereby contributing to complex alterations in sleep architecture. To comprehensively interpret how ASD phenotypes are recapitulated in mouse models and how they relate to clinical observations, it is essential to interactively examine both circadian activity and long-term sleep regulation under consistent experimental conditions. Furthermore, it is important to evaluate multiple aspects of sleep architecture, such as sleep duration, sleep fragmentation, and sleep latency (Wintler et al., 2020).

Recent advances in whole-exome sequencing have identified chromodomain helicase DNA-binding protein 8 (*CHD8*) as one of the high-penetrance ASD risk genes (Neale et al., 2012; O'Roak et al., 2012a,b; Study et al., 2014; Coll-Tané et al., 2021). Both individuals and mouse models with *CHD8*/*Chd8* mutations exhibit core ASD-like symptoms and distinct physical characteristics, including macrocephaly and severe gastrointestinal (GI) issues (O'Roak et al., 2012b; Talkowski et al., 2012; Barnard et al., 2015; Katayama et al., 2016). CHD8 is broadly expressed across the cortex and subcortical structures in both developing and adult human brains, with particularly high expression during the early and mid-fetal periods (9–21 weeks post-conception), which subsequently declines in adulthood (Bernier et al., 2014).

*CHD8* encodes a nuclear protein that belongs to the chromodomain-helicase-DNA binding protein (CHD) family. In humans, two predominant isoforms, *CHD8-L1* and *CHD8-L2*, have been identified. Both isoforms possess nuclear localization signals (NLSs) (Lu et al., 2021), a β-catenin binding site (β) that suppresses Wnt/β-catenin signaling via histone H1 recruitment (Nishiyama et al., 2012; Weissberg and Elliott, 2021), a pair of BRK domains that mediate chromatin interaction and bind to CCCTC-binding factor (CTCF), and an A-kinase anchoring protein (AKAP) domain (RII) (Shanks et al., 2012). In addition, both isoforms contain tandem chromodomains (C1 and C2) and a helicase domain. Notably, CHD8-L1 uniquely harbors a p53-binding domain that inhibits p53-mediated apoptosis (Nishiyama et al., 2009; Shanks et al., 2012; Weissberg and Elliott, 2021). Through this complex domain architecture, CHD8 plays a critical role in regulating transcription factor activity in neuronal progenitor cells, thereby contributing to neurodevelopmental processes (Barnard et al., 2015; Varghese et al., 2017; Derafshi et al., 2022). CHD8 has also been implicated in the transcriptional regulation of other ASD risk genes (Barnard et al., 2015; Varghese et al., 2017; Weissberg and Elliott, 2021).

A substantial proportion of individuals with *CHD8* mutations (approximately 67%) reported sleep-related problems, most commonly difficulty falling asleep and excessive daytime sleepiness (Matson et al., 2008; Mindell and Meltzer, 2008; Reynolds and Malow, 2011; Bernier et al., 2014; Barnard et al., 2015). Despite this, objective evaluations of sleep phenotypes in *CHD8* mutation carriers remain limited. In this study, we employed *Chd8* heterozygous knockout mice, since homozygous knockouts are embryonically lethal, to investigate their sleep-wake architecture and circadian activity. We observed increased REM sleep duration and episode number during the dark phase, the active period for nocturnal animals, along with a disruption in the diurnal regulation of NREM-to-REM transitions compared to littermate wild-type mice.

## 2 Material and methods

### 2.1 Animals

All experiments were performed using 9- to 12-week-old male and female littermates produced by wild-type (WT) crossed with heterozygous *Chd8* knockout mice (*Chd*8^+/−^). Since homozygous knockout mice are lethal, wild-type and heterozygous littermate animals were used for analysis. WT C57BL/6J mice used for mating were purchased from CLEA Japan Inc. For genotyping, tail tissue was collected from weaned mice. Genomic DNA was extracted by incubating tail samples in 50 mM NaOH at 95°C for 30 min, followed by neutralization with 1 M Tris-HCl (pH 8.0). The resulting DNA was used as a template for PCR. The primers used for genotyping were as follows: Chd8-WT-fw: 5′-GTTCACTCAGTAAATTTGTGTGCCTAC-3′; Chd8-mut-fw: 5′-GCAGCGCATCGCCTTCTATCGC-3′; Chd8-common-rv: 5′-GCTCCTATGTGTGCTGTCCTG-3′. The expected PCR product sizes were 443 bp for WT allele and 250 bp for the mutant allele. All animal experiments were approved by the University of Tsukuba Institutional Animal Care and Use Committee, thus following the guidelines of NIH. Mice were maintained in a home cage (17.3 × 38.5 × 14.8 cm, CLEA-Japan, Inc.) in an insulated chamber (45.7 × 50.8 × 85.4 cm), which was maintained at an ambient temperature of 23.5 ± 2.0°C under a 12-h light/dark cycle (9 am to 9 pm) with *ad libitum* access to food and water in accordance with institutional guidelines.

### 2.2 Wheel running and locomotion activity recording

Mice were single-housed in cages in which running wheels were equipped (MELQUEST, Japan) or above which IR sensors were equipped (Actimetrics, USA) to measure mouse activity. The cages were maintained in light-tight chambers illuminated with white light-emitting diodes (LEDs; 100 lx). For locomotor activity assessment, both male mice (WT: *n* = 15; *Chd*8^+/−^: *n* = 14) and female mice (WT: *n* = 4; *Chd*8^+/−^: *n* = 4) were used. Among them, a subset of male mice (WT: *n* = 8; *Chd*8^+/−^: *n* = 9) had previously undergone EEG recordings. For wheel-running activity assessment, only male mice were used (WT: *n* = 13; *Chd*8^+/−^: *n* = 11), including a subset (WT: *n* = 9; *Chd*8^+/−^: *n* = 8) that had also undergone EEG recordings before behavioral testing.

To confirm that EEG electrode implantation did not affect behavior, locomotor and wheel-running activity data were compared between EEG-implanted and intact mice. All activity data were collected in 1-min bins using ClockLab Data Collection software (Actimetrics, USA). The animals were first entrained to a 12/12 hr LD cycle (9:00 AM lights on, 9:00 PM lights off) for at least 7 days. Afterward, the mice were transferred to constant dark conditions (DD) (i.e., 24-h light off) and were recorded for at least 10 days to evaluate circadian activity. The circadian period and Qp value (a measure of rhythm robustness derived from chi-square periodogram analysis) were calculated by chi-square periodogram analysis using ClockLab Analysis 6 (Actimetrics, USA).

### 2.3 EEG/EMG implantation and recording

At 8 weeks of age, male mice (WT: *n* = 12; *Chd*8^+/−^: *n* = 11) and female mice (WT: *n* = 9; *Chd*8^+/−^: *n* = 8) were anesthetized with 1.5~2% isoflurane inhalation (Pfizer, United States) and fixed in a stereotaxic frame (Kopf Instruments, USA). Heights of the bregma and lambda was adjusted to be equal. Two EEG recording electrodes (stainless steel screws) connected to a 4-pin head mount (Cat#852-10-006-30-001101, Preci-dip, Switzerland) were implanted at AP +1.4 mm and −2.6 mm and ML −1.2 mm from the bregma. For EMG recording, two insulated silver wires connected to the same head mount were bilaterally inserted into the trapezius muscles. Two additional anchor screws were affixed to the skull to stabilize the implant. The entire assembly was fixed to the skull with dental cement (Cat#56849, 3M ESPE). The mouse skin was sutured using a sanitized silk thread (thread size, 6-0).

After 1 week of recovery, mice were habituated to the recording conditions for at least 5 days and then recorded for two consecutive 24-h periods. Averages of each time on two recording days were used as raw data, and data from all individual animals used in these studies were used to determine their sleep/wakefulness characteristics. EEG and EMG signals were amplified and filtered (EEG: 0.5–250 Hz, EMG: 16–250 Hz) using a multichannel amplifier (Cat#BAS-8102, Biotex, Japan) and commutator (Cat#SL-006, Biotex, Japan). A 50 Hz EEG notch filter was applied to reduce power line interference. The signals were digitized at a sampling rate of 128 Hz and recorded using Vital Recorder software (Kissei Comtec Co., Japan). Sleep stages were classified into wakefulness, non-rapid eye movement (NREM) sleep, and rapid eye movement (REM) sleep in 10-second epochs using the Sleep Analyzer Complex (SAC), a supervised deep learning-based model (Furutani et al., 2025). SAC extracted three types of features from EEG and EMG signals: (1) traditional spectral power and muscle tone; (2) signal irregularity, quantified by entropy; and (3) signal fractality, assessed by detrended fluctuation analysis. Each feature set was processed by a single-layer convolutional neural network. The resulting feature maps were concatenated and passed through two fully connected layers to predict sleep stages. Ground-truth labels for training dataset were generated by expert scorers based on prior studies (Mang and Franken, 2012), using EEG delta and theta power, theta regularity, and EMG tone to determine the dominant vigilance state in 25-second windows. These windows were shifted at 1-second increments to generate per-second labels. At the output layer, each epoch was scored as the stage with the highest softmax probability. After automated scoring by SAC, all epochs were manually reviewed and corrected as necessary. To evaluate sleep architecture, we measured the duration and number of episodes in each vigilance state, as well as the number of state transitions, as indicators of sleep fragmentation. EEG power spectra were calculated by fast Fourier transform (FFT). With a 128 Hz sampling rate and a 256-point FFT window, the frequency resolution was 0.5 Hz. Power in each frequency band was calculated every 10 seconds by averaging five consecutive 2-second FFT windows. Slow-wave activity (SWA) was defined as EEG power in the 0.5–4.0 Hz range during NREM sleep. A state transition matrix was constructed to assess transition probabilities between vigilance states. For example, the probability of transitioning from NREM to wakefulness was calculated by dividing the number of NREM → Wake transitions by the total number of transitions from NREM (i.e., NREM → Wake + NREM → REM).

### 2.4 Statistical analysis

No statistical methods were used to determine the sample size. The experiments were randomized. The investigators were not blinded to allocation during the experiments. All the results are presented as mean ± SEM with individual value plots, except for the daily profiles of sleep/wake duration and activity and distribution of REM latency. All statistical analyses were performed using GraphPad Prism 8.0. Type I error was set to 0.05. Shapiro-Wilk tests were performed to assess the normality of data. Brown-Forsythe tests were performed to assess homogeneity of variance. For comparisons between WT and *Chd8*^+/−^ mice, unpaired two-tailed *t*-tests were used. For comparison of time-series data between WT and *Chd8*^+/−^ mice, two-way ANOVA was performed, followed by the *post hoc* Sidak's test. For comparison of WT and *Chd8* mice in male and female mice, two-way ANOVA was performed, followed by the Fisher's Least Significant Difference (LSD) test. All statistical results, including mean, standard errors, *p* values, the main effect and interaction effect were indicated in the Supplementary Table S1. Statistical significances were indicated in figures as follows; ^*^ (*p* < 0.05), ^**^ (*p* < 0.01), ^***^ (*p* < 0.001), ^*****^ (*p* < 0.0001).

## 3 Results

### 3.1 Locomotor activity and wheel running activity

**Figure 1:**
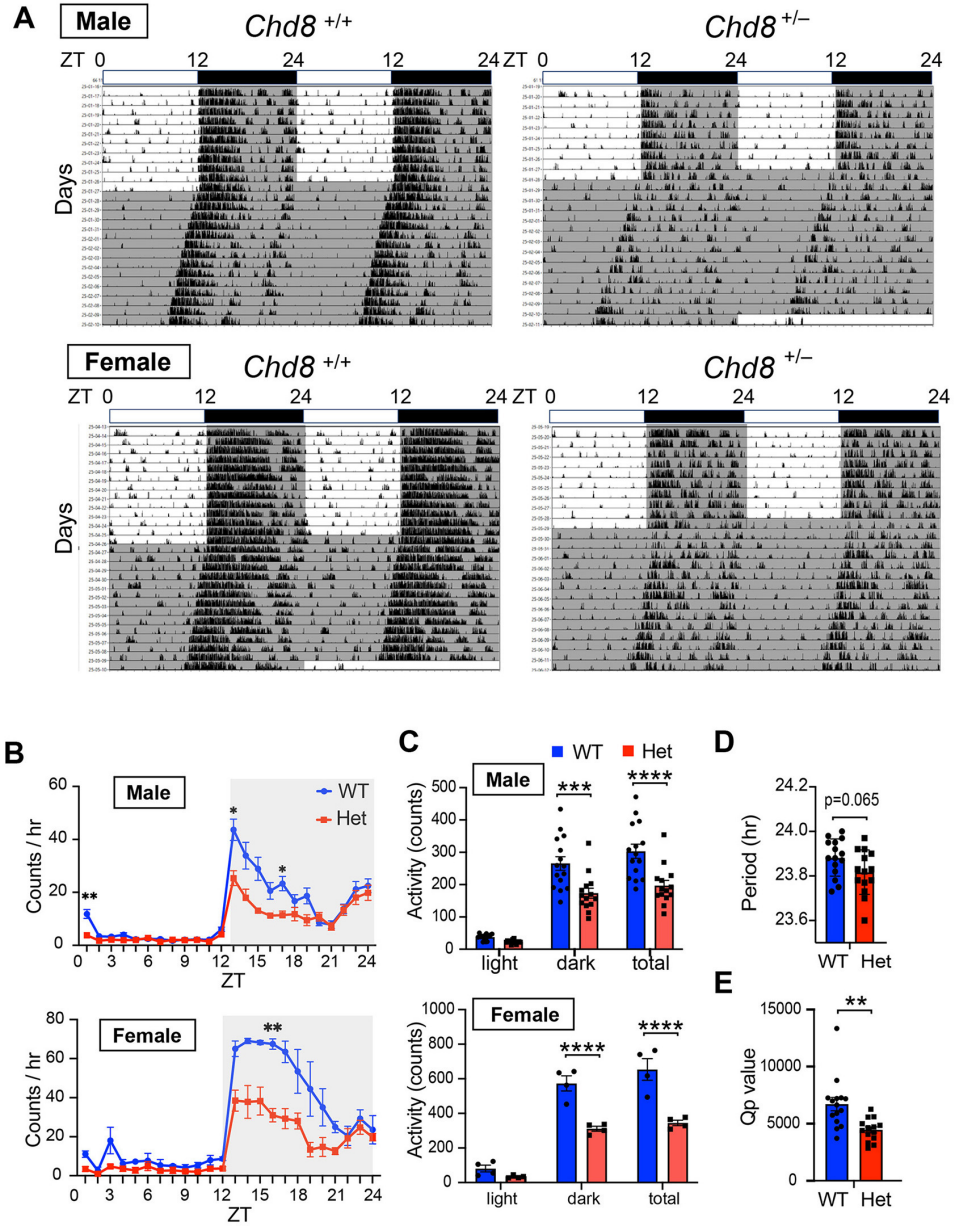
**(A)** Representative actogram of locomotor activity rhythms in male wild-type (*Chd*8^+/+^, upper left panel, *n* = 15), male heterozygous knockout mice (*Chd*8^+/−^, upper right panel, *n* = 14), female wildtype (*Chd*8^+/+^, bottom left panel, *n* = 4) and female heterozygous knockout mice (*Chd*8^+/−^, bottom right panel, *n* = 4) in LD and DD cycles. The bar on top depicts the lighting condition and zeitgeber time (ZT). The shaded area represents the period when the lights are off. **(B)** Activity profiles of *Chd8* mutant mice in LD cycle. Data are shown as mean with SEM (*n* = 15 for male *Chd*8^+/+^ and *n* = 14 for male *Chd*8^+/−^, *n* = 4 for female *Chd*8^+/+^ and *n* = 4 for female *Chd*8^+/−^). Two-way ANOVA, followed by Sidak's multiple comparison test was performed. Main effect (male WT vs. *Chd*8^+/−^); *p* = 0.0006, interacting effect (male WT vs. *Chd*8^+/−^); *p* < 0.0001, main effect (female WT vs. *Chd*8^+/−^); *p* = 0.0029, interacting effect (female WT vs. *Chd*8^+/−^); *p* = 0.0144. *P*-values by *post-hoc* analysis were indicated in the figures as asterisks. **(C)** Total activity level in 24 h in the LD and in 12 h of light or dark phase (*n* = 15 for male *Chd*8^+/+^ and *n* = 14 for male *Chd*8^+/−^, *n* = 4 for female *Chd*8^+/+^ and *n* = 4 for female *Chd*8^+/−^). Two-way ANOVA, followed by Sidak's multiple comparisons test, was performed. Main effect (male WT vs. *Chd*8^+/−^), *p* < 0.0001, interacting effect (male WT vs. *Chd*8^+/−^), *p* = 0.009, main effect (female WT vs. *Chd*8^+/−^), *p* < 0.0001, interacting effect (female WT vs. *Chd*8^+/−^), *p* = 0.0021. *P*-values by *post-hoc* analysis were indicated in the figures as asterisks. **(D)** Circadian period of locomotor activity rhythms in DD (*n* = 15 for male *Chd*8^+/+^ and *n* = 14 for male *Chd*8^+/−^; *p* = 0.0651 for comparison of period with unpaired *t*-test). **(E)** Qp values of locomotor activity rhythms in DD (*n* = 15 for male *Chd*8^+/+^ and *n* = 14 for male *Chd*8^+/−^; *p* = 0.0019 for comparison of Qp value with unpaired *t*-test).

We first assessed the locomotor activity of *Chd8* heterozygous knockout (*Chd*8^+/−^) and their WT littermates in their home cages. During the light-dark (LD) cycles, both *Chd*8^+/−^ mice and WT mice showed a typical nocturnal activity pattern, with increased locomotor activity during the dark phase (Figures 1A–C). However, both male and female *Chd*8^+/−^ mice exhibited significantly reduced activity during the early dark phase and lower total daily activity compared to WT mice (male WT vs. *Chd*8^+/−^ mice, dark phase activity: *p* = 0.0002; total activity: *p* < 0.0001; female WT vs. *Chd*8^+/−^ mice, dark phase activity: *p* < 0.0001 total activity: *p* < 0.0001, Figure 1C and Supplementary Table S1).

Because the free-running circadian period can influence the sleep-wake phase timing, we hypothesized that altered circadian periodicity might contribute to the sleep-onset difficulties and excessive daytime sleepiness often reported in individuals with ASD. To test this, we measured the free-running circadian period under constant darkness (DD). We found that male *Chd*8^+/−^ mice showed a trend toward a slightly shorter circadian period compared to WT mice (*p* = 0.0651, effect size = 0.71, mean difference= −0.06557, CI = −0.1335 to 0.0043; Figure 1D), although the absolute difference was minimal (approximately 5 min), suggesting that this alteration is unlikely to meaningfully affect the sleep phase. In female mice, no apparent trend toward a shorter period was observed; however, statistical analysis was not performed due to the limited sample size (*n* = 4 each genotype). *Chd*8^+/−^ male mice showed a significantly lower Qp value in the periodogram analysis in DD (male, *p* = 0.00144; Figure 1E). We concluded that *Chd*8^+/−^ mice have less robust circadian rhythms, likely due to the decreased activity during the subjective early dark period.

**Figure 2:**
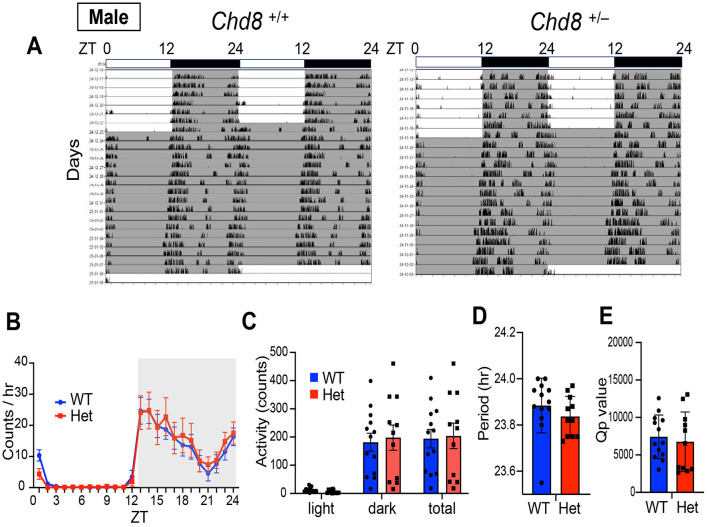
**(A)** Representative actogram of wheel-running activity rhythms in wild-type (male *Chd*8^+/+^, left panel, *n* = 13) and heterozygous knockout mice (male *Chd*8^+/−^, right panel, *n* = 11) in LD and DD cycles. The bar on top depicts the lighting condition and zeitgeber time (ZT). The shaded area represents the period when the lights are off. **(B)** Wheel-running activity profiles in LD cycle. Data are shown as mean with SEM (*n* = 13 for male *Chd*8^+/+^ and *n* = 11 for male *Chd*8^+/−^). **(C)** Total activity level in 24 h in the LD and in 12 h of the light or dark phase (*n* = 13 for male *Chd*8^+/+^ and *n* = 11 for male *Chd*8^+/−^). **(D)** Circadian period of locomotor activity rhythms in DD (*n* = 13 for male *Chd*8^+/+^ and *n* = 11 for male *Chd*8^+/−^; *p* = 0.2828 for comparison of period using unpaired *t*-test). **(E)** Qp values of locomotor activity rhythms in DD (*n* = 13 for male *Chd*8^+/+^ and *n* = 11 for male *Chd*8^+/−^; *p* = 0.6357 for comparison of Qp value with unpaired *t*-test).

We also monitored wheel running activity but found no significant differences between groups in terms of wheel running activity levels, circadian period and Qp values in male mice (male WT vs. *Chd*8^+/−^ mice, activity level: *p* = 0.9324, period: *p* = 0.2828, Qp values: *p* = 0.6357, Figures 2A–E). These results suggest that circadian activity may be manifested differently depending on the measurement used. Notably, because wheel running is a motivated behavior, the similar levels of activity observed between genotypes may reflect intact motivation in *Chd*8^+/−^ mice under these conditions.

### 3.2 Sleep and wake pattern and architecture

**Figure 3:**
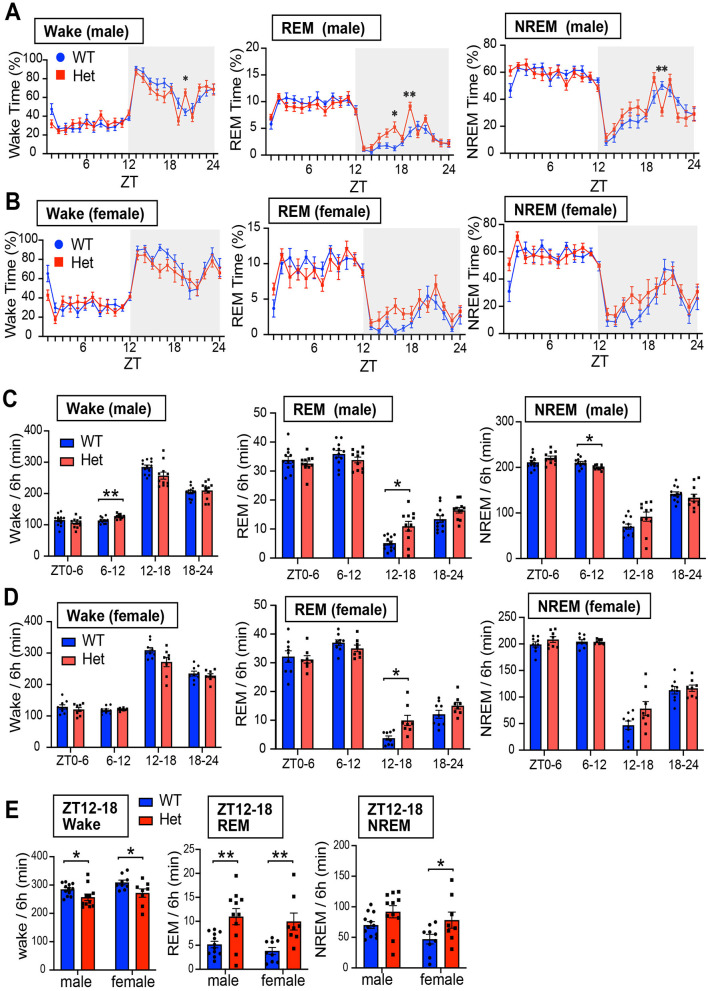
**(A)** Daily profiles of time duration of wakefulness, REM sleep, and NREM sleep in 1-h bin in LD cycle for male mice. Data are shown as mean with SEM (*n* = 12 for male *Chd*8^+/+^ and *n* = 11 for male *Chd*8^+/−^). Two-way ANOVA, followed by Sidak's multiple comparison test, was performed. Main effect for wake, REM and NREM duration were *p* = 0.3249, *p* = 0.106, and *p* = 0.4354. Interacting effect (male WT vs. *Chd*8^+/−^) for wake, REM and NREM duration were *p* = 0.0011, *p* = 0.0095, and *p* = 0.0267. *P*-values by *post-hoc* analysis were indicated in the figures as asterisks. **(B)** Daily profiles of time duration of wakefulness, REM sleep, and NREM sleep in 1-h bin in LD cycle for female mice. Data are shown as mean with SEM (*n* = 9 for female *Chd*8^+/+^ and *n* = 8 for female *Chd*8^+/−^). Two-way ANOVA, followed by Sidak's multiple comparison test, was performed. Main effect (female WT vs. *Chd*8^+/−^) for Wake, REM, and NERM duration were *p* = 0.0648, *p* = 0.155, and *p* = 0.0495. Interacting effect (female WT vs. *Chd*8^+/−^) for Wake, REM, and NERM duration were *p* = 0.3468, *p* = 0.1381, and *p* = 0.3499. **(C)** Total duration of wakefulness, REM sleep, and NREM sleep in 6 h for male mice. Data are shown as mean with SEM (*n* = 12 for male *Chd*8^+/+^ and *n* = 11 for male *Chd*8^+/−^). Two-way ANOVA, followed by Sidak's multiple comparison test, was performed. Main effects (male WT vs. *Chd*8^+/−^) in Wake REM sleep, and NREM sleep duration were *p* = 0.3248, *p* = 0.1055 and *p* = 0.435. Interacting effects were *p* = 0.00198, *p* = 0.0023, *p* = 0.0233. P-values by *post-hoc* analysis were indicated in the figures as asterisks (Wake ZT6-12 in male: *p* = 0.0064; REM sleep ZT12-18; NREM ZT6-12 in male: *p* = 0.0223: *p* = 0.0263). **(D)** Total duration of wakefulness, REM sleep, and NREM sleep in 6 h for female mice. Data are shown as mean with SEM (*n* = 9 for female *Chd*8^+/+^ and *n* = 8 for female *Chd*8^+/−^). Two-way ANOVA, followed by Sidak's multiple comparison test, was performed. Main effects (female WT vs. female *Chd*8^+/−^) for Wake, REM, and NREM duration were *p* = 0.0648, *p* = 0.1505, and 0.0681. Interacting effects were *p* = 0.071, *p* = 0.0149, and *p* = 0.1028. **(E)** Duration of wakefulness, REM sleep, and NREM sleep in ZT12-18 for male and female mice. Data are shown as mean with SEM (*n* = 12 for male *Chd*8^+/+^, *n* = 11 for male, *Chd*8^+/−^*n* = 9 for female *Chd*8^+/+^, and *n* = 8 for female *Chd*8^+/−^). Two-way ANOVA, followed by Fisher's LSD test, was performed. Main effects (WT vs. *Chd*8^+/−^) for Wake, REM, and NREM duration were *p* = 0.0032, *p* < 0.0001, and 0.0062. Interacting effects (WT vs. *Chd*8^+/−^ x male vs. female) were *p* = 0.1028, *p* = 0.6335, and *p* = 0.9011. *P*-values by *post-hoc* analysis were indicated in the figures as asterisks.

**Figure 4:**
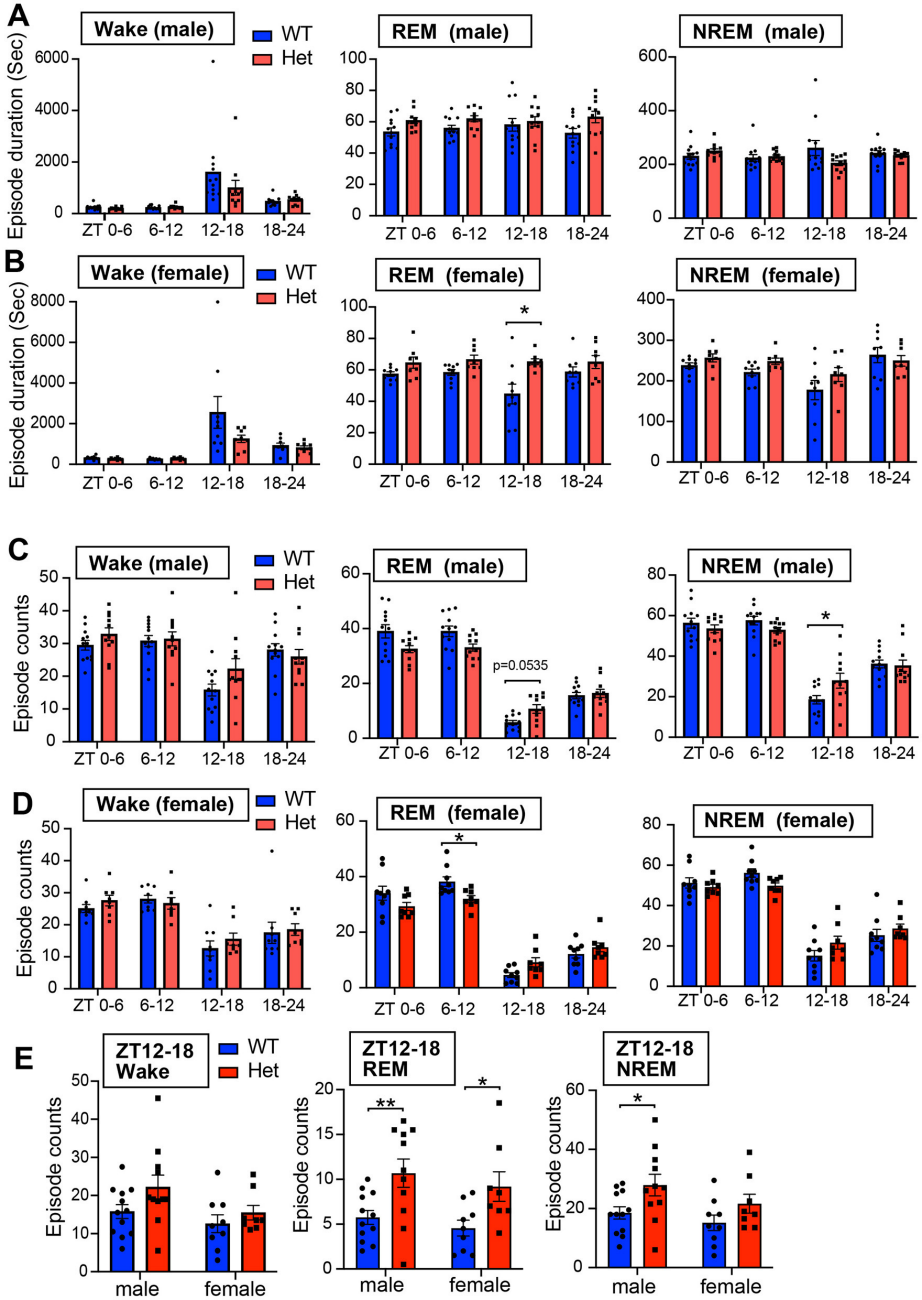
**(A)** Episode duration of wakefulness, REM sleep, and NREM sleep in 6 h for male mice. Data are shown as mean with SEM (*n* = 12 for male *Chd*8^+/+^ and *n* = 11 for male *Chd*8^+/−^). Two-way ANOVA, followed by Sidak's multiple comparison test, was performed. Main effects (male WT vs. *Chd*8^+/−^) in Wake REM sleep, and NREM sleep duration were *p* = 0.3121, *p* = 0.0391 and *p* = 0.3813. Interacting effects were *p* = 0.2323, *p* = 0.3797, and *p* = 0.0217. **(B)** Episode duration of wakefulness, REM sleep, and NREM sleep in 6 h for female mice. Data are shown as mean with SEM (*n* = 9 for female *Chd*8^+/+^ and *n* = 8 for female *Chd*8^+/−^). Two-way ANOVA, followed by Sidak's multiple comparison test, was performed. Main effects (female WT vs. female *Chd*8^+/−^) for Wake, REM, and NREM duration were *p* = 0.1485, *p* = 0.0038, and *p* = 0.1565. Interacting effects were *p* = 0.0927, *p* = 0.0993, and *p* = 0.2674. *P*-values by *post hoc* analysis were indicated in the figures as asterisks (REM ZT12-18, *p* = 0.0452). **(C)** Episode number of wakefulness, REM sleep, and NREM sleep in 6 h for male mice. Data are shown as mean with SEM (*n* = 12 for male *Chd*8^+/+^ and *n* = 11 for male *Chd*8^+/−^). Two-way ANOVA, followed by Sidak's multiple comparison test, was performed. Main effects (male WT vs. *Chd*8^+/−^) in Wake REM sleep, and NREM sleep duration were *p* = 0.399, *p* = 0.2865, and *p* = 0.9225. Interacting effects were *p* = 0.0125, *p* < 0.0001, and *p* = 0.0019. *P*-values by *post hoc* analysis were indicated in the figures as asterisks (NREM, ZT12-18, *p* = 0.025). **(D)** Episode number of wakefulness, REM sleep, and NREM sleep in 6 h for female mice. Data are shown as mean with SEM (*n* = 9 for female *Chd*8^+/+^ and *n* = 8 for female *Chd*8^+/−^). Two-way ANOVA, followed by Sidak's multiple comparison test, was performed. Main effects (female WT vs. female *Chd*8^+/−^) for Wake, REM, and NREM duration were *p* = 0.5995, *p* = 0.5233, and *p* = 0.8926. Interacting effects were *p* = 0.3943, *p* = 0.0007, and *p* = 0.0051. *P*-values by *post hoc* analysis were indicated in the figures as asterisks (REM ZT6-12, *p* = 0.0381). **(E)** Episode number of each sleep state in ZT12-18 for male and female mice. Data are shown as mean with SEM (*n* = 12 for male *Chd*8^+/+^, *n* = 11 for male, *Chd*8^+/−^*n* = 9 for female *Chd*8^+/+^, and *n* = 8 for female *Chd*8^+/−^). Two-way ANOVA, followed by Fisher's LSD test, was performed. Main effects (WT vs. *Chd*8^+/−^) for Wake, REM, and NREM duration were *p* = 0.0657, *p* = 0.0006, and 0.0113. Interacting effects (WT vs. *Chd*8^+/−^ x male vs. female) were *p* = 0.4749, *p* = 0.9064, and *p* = 0.6224. *P*-values by *post hoc* analysis were indicated in the figures as asterisks.

We next investigated sleep-wake architecture in both male and female mice using EEG and EMG recordings. We evaluated sleep duration, sleep fragmentation, REM sleep latency, and EEG power spectra (Figures 3, 4, and Supplementary Figure S1). Consistent with reduced locomotor activity observed during the early dark phase in *Chd*8^+/−^ mice (Figure 1B), both male and female *Chd*8^+/−^ mice exhibited a trend toward decreased wakefulness and increased sleep time (NREM and REM sleep) during the night phase (Figures 3A, B), potentially modeling the excessive daytime sleepiness reported in human patient with ASD. In particular, REM sleep duration was significantly increased during ZT12-18 in both sexes (REM duration in ZT12-18, male: *p* = 0.0263, female: *p* = 0.0409). The distribution of vigilance states during ZT12-18 was comparable between male and female mice (Figure 3E, wakefulness, male: *p* = 0.047, female: *p* = 0.0217; REM sleep, male: *p* = 0.0014, female: *p* = 0.0034; NREM sleep, male: *p* = 0.0757, female: *p* = 0.0298). However, we also observed sex-specific alterations. During the second half of the resting period (ZT6-12), only male *Chd*8^+/−^ mice showed increased wakefulness and reduced NREM sleep (Figure 3C, wake: *p* = 0.0064; NREM: *p* = 0.0223). In addition, while WT mice typically showed a napping period in the middle of the dark phase (approximately ZT19-21), this pattern was disrupted in the *Chd*8^+/−^ males. Specifically, male *Chd*8^+/−^ mice showed altered vigilance states during this window, with significant changes at several time points (Figure 3A, wake at ZT20: *p* = 0.0224; REM sleep at ZT17: *p* = 0.0492; REM sleep at ZT19: *p* = 0.0098; NREM sleep at ZT20: *p* = 0.0059).

For evaluating sleep fragmentation, we analyzed the duration of each episode and found no significant differences between genotypes in NREM sleep and wake stages in both male and female mice (Figures 4A, B). However, female *Chd*8^+/−^ mice showed significantly longer REM sleep episodes during ZT12-18 (*p* = 0.0452, Figures 4A, B). In contrast to the episode duration, the number of REM sleep episodes was consistently affected across sexes: reduced in the light phase and increased in the dark phase in *Chd*8^+/−^ mice (Figures 4C, D, and Supplementary Figure S2A). Aligned with the longer REM duration at night, both male and female mice showed an increased number of REM episodes in ZT12-18 (male: *p* = 0.005; female: *p* = 0.0211, Figure 4E). The number of wake and NREM bouts also exhibited a similar tendency across sexes in ZT12-18 (Figure 4E). These findings indicate that the normal daily fluctuation in REM sleep was attenuated in *Chd*8^+/−^ mice.

**Figure 5:**
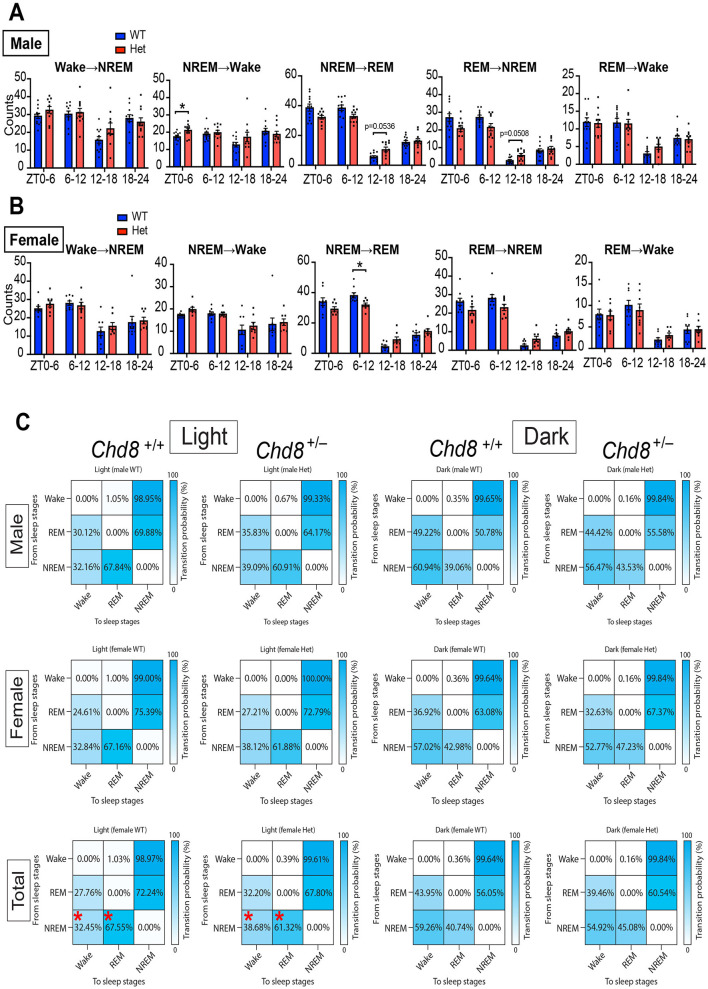
**(A)** Sleep state transition in 6 h for male mice. Data are shown as mean with SEM (*n* = 12 for male *Chd*8^+/+^ and *n* = 11 for male *Chd*8^+/−^). Two-way ANOVA, followed by Sidak's multiple comparison test, was performed. Main effects (male WT vs. *Chd*8^+/−^) in each transition were *p* = 0.3821 (W→ NREM), *p* = 0.2839 (NREM→ W), *p* = 0.2982 (NREM→ REM), *p* = 0.1675 (REM→ NREM), and *p* = 0.8189 (REM→ Wake). Interacting effects were *p* = 0.0123 (W→ NREM), *p* = 0.0226 (NREM→ W), *p* < 0.0001 (NREM→ REM), *p* = 0.0005 (REM→ NREM), and *p* = 0.266 (REM→ Wake). *P*-values by *post-hoc* analysis were indicated in the figures as asterisks. **(B)** Episode duration of wakefulness, REM sleep, and NREM sleep in 6 h for female mice. Data are shown as mean with SEM (*n* = 9 for female *Chd*8^+/+^ and *n* = 8 for female *Chd*8^+/−^). Two-way ANOVA, followed by Sidak's multiple comparison test, was performed. Main effects (female WT vs. *Chd*8^+/−^) in each transition were *p* = 0.5995 (W→ NREM), *p* = 0.4074 (NREM→ W), *p* = 0.5233 (NREM→ REM), *p* = 0.5059 (REM→ NREM), and *p* = 0.9613 (REM→ Wake). Interacting effects were *p* = 0.3943 (W→ NREM), *p* = 0.7262 (NREM→ W), *p* = 0.0007 (NREM→ REM), *p* = 0.0025 (REM→ NREM) and *p* = 0.4769 (REM→ Wake). *P*-values by *post-hoc* analysis were indicated in the figures as asterisks. **(C)** State-to-state transition matrix in light and night phase for male, female and total (mixed male and female) mice. The row shows a pre-state of transition. The column shows a post-state of transition. Data are shown as mean (*n* = 12 for male *Chd*8^+/+^, *n* = 11 for male *Chd*8^+/−^, *n* = 9 for female *Chd*8^+/+^ and *n* = 8 for female *Chd*8^+/−^). Two-way ANOVA, followed by Sidak's multiple comparisons test, was performed. Main effects (WT vs. *Chd*8^+/−^) were all *p* > 0.9999. Interacting effects were *p* = 0.0031 (light, male), *p* = 0.3163 (dark, male), *p* = 0.1476 (light, female), *p* = 0.6314 (dark, female), *p* = 0.0002 (light, total), and *p* = 0.1487 (dark, total). *P*-values by *post-hoc* analysis were indicated in the figures as asterisks.

To further evaluate fragmentation, we quantified vigilance state transitions in 6-h bins. Since transitions from wake to REM sleep were extremely rare, only five transition types were analyzed (Figures 5A, B, and Supplementary Figure S2B). These transition patterns were similar across sexes, indicating that the observed effects were sex-independent. In both male and female *Chd*8^+/−^ mice, transitions between NREM and REM sleep showed a distinct circadian pattern: reduced during the light phase and increased during the dark phase. These results suggest dysregulation of the circadian control of the NREM–REM cycle. Transition probability matrices further summarized this shared phenotype across sexes (mixed male and female WT vs. *Chd*8^+/−^, NREM → Wake, *p* = 0.0232; NREM → REM, *p* = 0.0232, Figure 5C).

**Figure 6:**
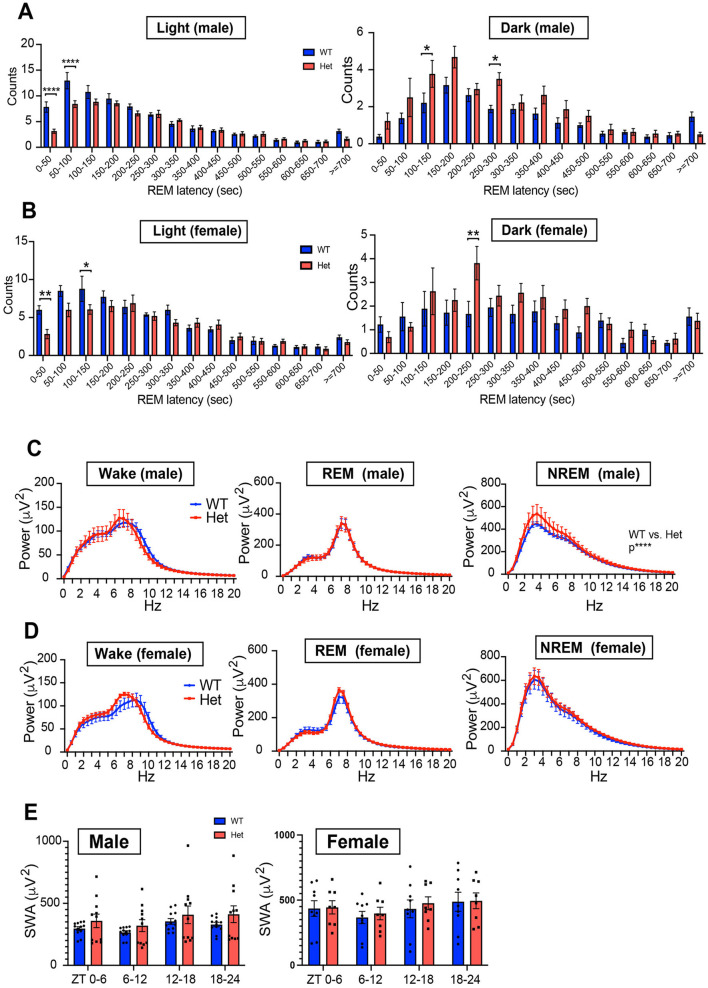
**(A)** Distribution of REM sleep latency from NREM sleep onset during the light phase for male mice (*n* = 12 for male *Chd*8^+/+^ and *n* = 11 for *Chd*8^+/−^). Two-way ANOVA, followed by Sidak's multiple comparison test, was performed. Main effects (male WT vs. *Chd*8^+/−^) during light and night phases were *p* = 0.0002 and *p* < 0.0001. Interacting effects were *p* < 0.0001 and *p* = 0.0417. P-values by *post-hoc* analysis were indicated in the figures as asterisks. **(B)** Distribution of REM sleep latency from NREM sleep onset during the light phase for female mice (*n* = 9 for female *Chd*8^+/+^ and *n* = 8 for *Chd*8^+/−^). Two-way ANOVA, followed by Sidak's multiple comparison test, was performed. Main effects (female WT vs. *Chd*8^+/−^) during light and night phases were *p* = 0.0057 and *p* = 0.0093. Interacting effects were *p* = 0.0074 and *p* = 0.1673. *P*-values by *post-hoc* analysis were indicated in the figures as asterisks. **(C)** EEG power spectrum for each sleep stage for male mice (*n* = 12 for male *Chd*8^+/+^ and *n* = 11 for *Chd*8^+/−^). Two-way ANOVA, followed by Sidak's multiple comparison test, was performed. Main effects (male WT vs. *Chd*8^+/−^) in Wake, REM and NREM sleep were *p* = 0.4403, *p* = 9139 and *p* < 0.0001. Interacting effects were *p* > 0.9999, *p* = 0.9999, and *p* = 0.9897. **(D)** EEG power spectrum for each sleep stage for female mice (*n* = 9 for female *Chd*8^+/+^ and *n* = 8 for *Chd*8^+/−^). Two-way ANOVA, followed by Sidak's multiple comparison test, was performed. Main effects (male WT vs. *Chd*8^+/−^) in Wake, REM and NREM sleep were *p* = 0.2773, *p* = 0.2594 and *p* = 0.0777. Interacting effects were *p* = 0.5144, *p* = 0.9821, and *p* > 0.9999. **(E)** Slow Wave Activity (SWA) in NREM sleep in 6 h for male and female mice. Data are shown as mean with SEM (*n* = 12 for male *Chd*8^+/+^, *n* = 11 for male *Chd*8^+/−^, *n* = 9 for female *Chd*8^+/+^ and *n* = 8 for female *Chd*8^+/−^). Two-way ANOVA, followed by Sidak's multiple comparison test, was performed. Main effects (male WT vs. *Chd*8^+/−^) in male and female mice were *p* = 0.3008 and *p* = 0.7908. Interacting effects were *p* = 0.4341 and *p* = 0.6176.

We further assessed the distribution of REM episodes based on REM latency (time from NREM sleep onset to REM sleep onset, Figures 6A, B). During the light phase, both male and female *Chd*8^+/−^ mice exhibited fewer REM sleep episodes with short REM latency, specifically within the latency ranges of 0–50 second (*p* < 0.0001) and 50–100 second (*p* < 0.0001) in male and that of 0–50 second (*p* = 0.0055) and 100–150 second (*p* = 0.0343) in female mice. This suggests that *Chd*8^+/−^ mice are less likely to enter REM sleep during the daytime, consistent with the decreased NREM-REM transitions (Figures 5A, B). In the dark phase, *Chd*8^+/−^ mice showed a greater number of REM sleep episodes with intermediate latencies (REM latency of 100–150 seconds in male mice: *p* = 0.0459; 250–300 seconds: *p* = 0.0318; 200–250 seconds in female mice: *p* = 0.0067), although the overall distribution pattern remained similar to that observed in WT mice (Figures 6A, B). This indicates that while REM timing is shifted, REM occurrence is preserved or even enhanced during the active phase in mutant mice. Furthermore, we analyzed EEG spectral power during each vigilance state. No significant differences were observed in EEG spectral power during wakefulness or REM sleep. However, the EEG power spectrum in NREM sleep was significantly altered in male *Chd*8^+/−^ mice compared to WT mice (main effect in two-way ANOVA, WT vs. *Chd*8^+/−^: *p* < 0.0001, Figure 6C), while this effect was not observed in the female mice (Figure 6D). Time-dependent analysis of slow wave activity in NREM sleep in 6-h bins revealed no significant interaction between time and genotype (Figure 6E), suggesting that the increased sleep pressure in male *Chd*8^+/−^ mice may be persistent rather than phase-specific.

## 4 Discussion

ASD is a neurodevelopmental disorder characterized by deficits in social communication and interaction and repetitive and restricted behaviors. Individuals with *CHD8* mutations exhibit not only core ASD symptoms but also distinct physiological alterations, such as macrocephaly (Wintler et al., 2020). A previous study reported that mice with heterozygous *Chd8* gene deletion exhibited similar physiological and behavioral changes, including macrocephaly, excessive anxiety, repetitive behaviors, and abnormal social behaviors (Katayama et al., 2016). Approximately 67% of individuals with *CHD8* mutations report severe sleep disturbances (Wintler et al., 2020). Compared to individuals with mutations in other ASD risk genes, those with *CHD8* mutations tend to experience more specific sleep problems, such as difficulty initiating sleep and excessive daytime sleepiness (Coll-Tané et al., 2021). However, to our knowledge, no previous study has comprehensively examined sleep architecture and circadian characteristics in *Chd8* knockout mice as an ASD model animal.

In this study, we found that *Chd*8^+/−^ mice exhibited reduced locomotor activity in their home cages during the early dark phase compared with WT mice (Figure 1), despite no alterations in entrainment. It is unlikely that the reduced activity was due to motor dysfunction, as previous studies have shown that the moving distance of the mutant mice in the open field test was comparable to that of littermate WT mice (Katayama et al., 2016). We also observed a decreased wake duration and increased sleep duration in the early dark phase, which aligns with the circadian activity results. These results suggest that the circadian output from the central clock, or the oscillation of the clock itself, may be weakened in the *Chd*8^+/−^ mice. Notably, mild melatonin treatment has been reported as an effective intervention for improving sleep problems in individuals with ASD (Malow et al., 2012; Buckley et al., 2020), potentially through enhancement of central clock function. Consistent with findings from human studies, melatonin administration in an ASD mouse model with a *Ctnnd2* mutation was shown to restore REM sleep deficits and alleviate ASD-like behaviors (Xu et al., 2023).

The most prominently affected aspect of sleep architecture in both male and female *Chd*8^+/−^ mice is the daily profile of REM sleep, which is typically regulated in a circadian manner and strongly suppressed during the night in WT mice. In contrast, *Chd*8^+/−^ mice exhibited a reduced number of REM sleep episodes during the light phase, whereas the total REM sleep duration and episode number were increased in the dark phase (Figures 3A, B, 4C, D). We hypothesize that the REM sleep amount during the light phase (resting period for mice) may be compromised in *Chd*8^+/−^ mice, potentially leading to compensatory REM sleep prolongation during the dark phase. These findings suggest that while the homeostatic regulation of REM sleep is preserved, its circadian modulation may be disrupted in *Chd*8^+/−^ mice. Notably, *Chd*8^+/−^ mice displayed fewer REM episodes with short latency (< 150 seconds) during the light phase (Figures 6A, B). A similar phenotype of longer REM sleep latency has been reported in human ASD patients (Kawai et al., 2022), suggesting that our autism model partially recapitulates the sleep phenotype in ASD patients. In the same study, the authors also reported that ASD patients exhibited an increased ratio of N3 stage (slow-wave sleep) to total sleep, which was positively correlated with the severity of core ASD symptoms. The elevated EEG power of the low-frequency band in NREM sleep in male *Chd*8^+/−^ mice (Figure 6C) may be associated with the enhancement of slow-wave activity in human ASD. There are few studies examining the EEG power spectrum in ASD model animals. In *Shank3* mutant mice, the delta power in NREM sleep was decreased, and the authors suggested decreased sleep quality (Ingiosi et al., 2019). *Scn1a* mutant mice serving as a Dravet Syndrome model with autism spectrum also showed a reduction in delta power (Kalume et al., 2015). On the other hand, in human ASD patients, excessive power of delta and theta band waves were reported (Wang et al., 2013). In general, an increase in delta power is thought to be associated with sleepiness in mice (Funato et al., 2016). However, our results from female mice suggested that increased sleep time during the nighttime (daytime for humans) was not associated with the altered EEG power because female mice exhibited an intact EEG spectrum (Figure 6D). Thus, the phenotype in EEG power is complex and needs further comprehensive analysis.

Most ASD mouse models with sleep problems exhibit increased wakefulness or reduced sleep; however, some ASD mouse models, including ours, have shown increased REM sleep. For example, a study reported that mice with the *Csnk1e tau* mutation exhibited increased REM sleep amount during the dark phase compared with *Csnk1e* null mutant or WT mice (Zhou et al., 2014). Similarly, *Shank3*-mutated mice displayed increased REM sleep during the light phase between postnatal days 23 to 59, and increased REM sleep during the dark phase at postnatal day 29 (Medina et al., 2022). These studies suggest that various factors, such as differences in mutated genes and the developmental stages of the mice, can affect sleep traits. Therefore, the impact of age or developmental stage on sleep phenotype in *Chd*8^+/−^ mice remains to be elucidated. The mechanism underlying the altered sleep architecture found in *Chd*8^+/−^ mice also remains largely unknown. Melanin-concentrating hormone (MCH), produced by neurons in the lateral hypothalamus, plays a critical role in the initiation and maintenance of REM sleep (Clément et al., 2012; Luppi et al., 2013; Izawa et al., 2019). Interestingly, bilateral silencing of MCH-producing neurons has been shown to increase the number of REM sleep bouts during the dark phase, resulting in a hypnogram similar to that observed in our findings (Clément et al., 2012) (Figures 3A, B).

Our study on the circadian and sleep phenotypes of *Chd*8^+/−^ mice contributes to understanding how genetically driven ASD can affect sleep behavior in humans and may provide valuable clinical insights into sleep disturbances associated with ASD.

## Data Availability

The original contributions presented in the study are included in the article/Supplementary material, further inquiries can be directed to the corresponding authors.

## Supplementary material

Supplementary Figure S1 **(A)** Schedule of EEG recording and positions of electrodes. **(B, C)** Representative EEG traces and hypnograms recorded from wild type (*Chd*8^+/+^) and heterozygous knockout mice (*Chd*8^+/−^) for male **(B)** and female **(C)**.

Supplementary Figure S2 **(A)** Relative episode number of each vigilance state in 6-hr bin for male mice. Data are shown as mean with SEM (*n* = 12 for male *Chd*8^+/+^ and *n* = 11 for male *Chd*8^+/−^). The episode number was divided by the daily total episodes. Two-way ANOVA, followed by Sidak's multiple comparisons test, was performed. Main effects for wake, REM, and NREM were *p* = 0.5769, *p* = 0.6103, and *p* = 0.7713. Interacting effects (male WT vs. *Chd*8^+/−^) for wake, REM, and NREM were *p* = 0.0034, *p* = 0.0061, and *p* = 0.9485. *P*-values by *post hoc* analysis were indicated in the figures as asterisks. **(B)** Relative episode number of each vigilance state in 6-h bin for female mice. Data are shown as mean with SEM (*n* = 9 for female *Chd*8^+/+^ and *n* = 8 for female *Chd*8^+/−^). The episode number was divided by the daily total episodes. Two-way ANOVA, followed by Sidak's multiple comparisons test, was performed. Main effects (female WT vs. *Chd*8^+/−^) for Wake, REM, and NERM were *p* = 0.9093, *p* = 0.9477, and *p* = 0.8915. Interacting effects (female WT vs. *Chd*8^+/−^) for Wake, REM, and NERM were *p* = 0.0239, *p* = 0.0361, and *p* = 0.9868. **(C)** Relative number of each state transition in 6-hr bin for male mice. Data are shown as mean with SEM (*n* = 12 for male *Chd*8^+/+^ and *n* = 11 for male *Chd*8^+/−^). The transition number for each state-to-state was divided by the daily total transition. Two-way ANOVA, followed by Sidak's multiple comparisons test, was performed. Main effects (male WT vs. *Chd*8^+/−^) in each transition were *p* = 0.5384 (W → NREM), *p* = 0.7734 (NREM → W), *p* = 0.6552 (NREM → REM), *p* = 0.5623 (REM → NREM), and *p* = 0.7195 (REM → Wake). Interacting effects were *p* = 0.0034 (W → NREM), *p* = 0.0212 (NREM → W), *p* = 0.0057 (NREM → REM), *p* = 0.0057 (REM → NREM), and *p* = 0.9292 (REM → Wake). *P*-values by *post hoc* analysis were indicated in the figures as asterisks. **(D)** Relative number of each state transition in 6-h bin for female mice. Data are shown as mean with SEM (*n* = 9 for female *Chd*8^+/+^ and *n* = 8 for female *Chd*8^+/−^). The transition number for each state-to-state was divided by the daily total transition. Two-way ANOVA, followed by Sidak's multiple comparison test, was performed. Main effects (male WT vs. *Chd*8^+/−^) in each transition were *p* = 0.9093 (W → NREM), *p* = 0.9931 (NREM → W), *p* = 0.9477 (NREM → REM), *p* = 0.8889 (REM → NREM), and *p* = 0.1706 (REM → Wake). Interacting effects were *p* = 0.0239 (W → NREM), *p* = 0.1003 (NREM → W), *p* = 0.0361 (NREM → REM), *p* = 0.0421 (REM → NREM) and *p* = 0.9868 (REM → Wake). *P*-values by *post hoc* analysis were indicated in the figures as asterisks.

Supplementary Table S1 Means with SEM, main effect and interaction effect and *p* values by statistical analysis were shown.
