# Supplementary figures and images for: Circadian activity and sleep architecture in autism spectrum disorder mouse model with Chd8 mutation
Source: Front Sleep. 2025 Aug 6;4:1614100. doi: 10.3389/frsle.2025.1614100 (PMC12713839; doi:10.3389/frsle.2025.1614100)

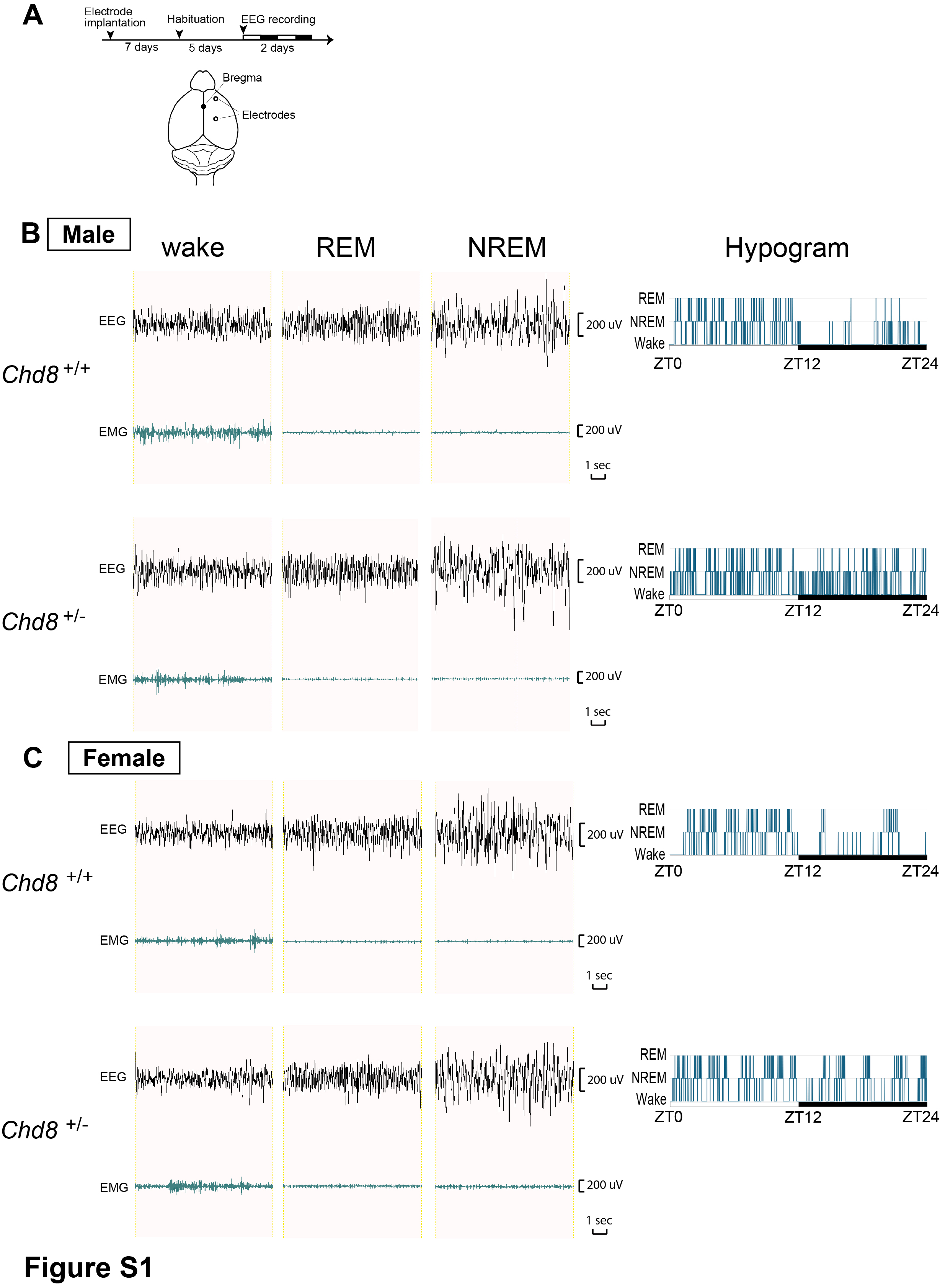

Supplement: Supplementary Figure S1 — (A) Schedule of EEG recording and positions of electrodes. (B, C) Representative EEG traces and hypnograms recorded from wild type (Chd8+/+) and heterozygous knockout mice (Chd8+/−) for male (B) and female (C). [file Image_1.jpeg]

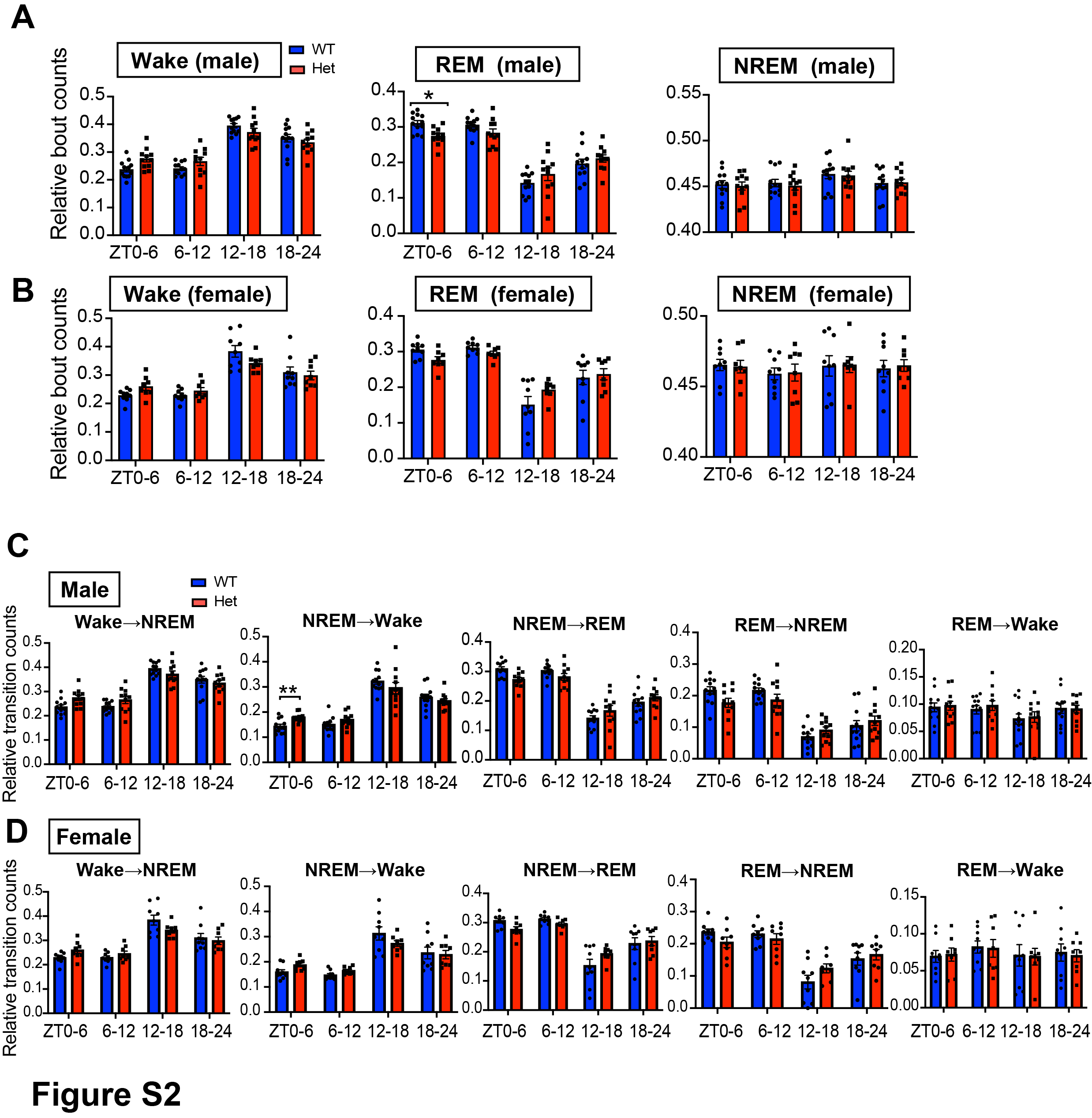

Supplement: Supplementary Figure S2 — (A) Relative episode number of each vigilance state in 6-hr bin for male mice. Data are shown as mean with SEM (n = 12 for male Chd8+/+ and n = 11 for male Chd8+/−). The episode number was divided by the daily total episodes. Two-way ANOVA, followed by Sidak's multiple comparisons test, was performed. Main effects for wake, REM, and NREM were p = 0.5769, p = 0.6103, and p = 0.7713. Interacting effects (male WT vs. Chd8+/−) for wake, REM, and NREM were p = 0.0034, p = 0.0061, and p = 0.9485. P-values by post hoc analysis were indicated in the figures as asterisks. (B) Relative episode number of each vigilance state in 6-h bin for female mice. Data are shown as mean with SEM (n = 9 for female Chd8+/+ and n = 8 for female Chd8+/−). The episode number was divided by the daily total episodes. Two-way ANOVA, followed by Sidak's multiple comparisons test, was performed. Main effects (female WT vs. Chd8+/−) for Wake, REM, and NERM were p = 0.9093, p = 0.9477, and p = 0.8915. Interacting effects (female WT vs. Chd8+/−) for Wake, REM, and NERM were p = 0.0239, p = 0.0361, and p = 0.9868. (C) Relative number of each state transition in 6-hr bin for male mice. Data are shown as mean with SEM (n = 12 for male Chd8+/+ and n = 11 for male Chd8+/−). The transition number for each state-to-state was divided by the daily total transition. Two-way ANOVA, followed by Sidak's multiple comparisons test, was performed. Main effects (male WT vs. Chd8+/−) in each transition were p = 0.5384 (W → NREM), p = 0.7734 (NREM → W), p = 0.6552 (NREM → REM), p = 0.5623 (REM → NREM), and p = 0.7195 (REM → Wake). Interacting effects were p = 0.0034 (W → NREM), p = 0.0212 (NREM → W), p = 0.0057 (NREM → REM), p = 0.0057 (REM → NREM), and p = 0.9292 (REM → Wake). P-values by post hoc analysis were indicated in the figures as asterisks. (D) Relative number of each state transition in 6-h bin for female mice. Data are shown as mean with SEM (n = 9 for female Chd8+/+ and n = 8 for female Chd8+/−). The transi [file Image_2.jpg]
